# Effects of Exergame with Biofeedback Training on Functional Status, Cognition, and Quality of Life in Outpatients with Polyneuropathies: A Longitudinal Pilot Study

**DOI:** 10.3390/brainsci16010045

**Published:** 2025-12-29

**Authors:** Francesco Zanatta, Daniela Mancini, Patrizia Steca, Monica Panigazzi, Elena Prestifilippo, Cesare Grilli, Marco D’Addario, Antonia Pierobon, Marina Maffoni

**Affiliations:** 1 Department of Psychology, University of Milano-Bicocca, 20126 Milan, Italy; francesco.zanatta@unimib.it (F.Z.); patrizia.steca@unimib.it (P.S.); marco.daddario@unimib.it (M.D.); 2 Psychology Unit of Montescano Institute, Istituti Clinici Scientifici Maugeri IRCCS, 27040 Montescano, Italy; daniela.mancini@virgilio.it (D.M.); marina.maffoni@icsmaugeri.it (M.M.); 3 Occupational Physiatry and Ergonomics Unit of Montescano Insitute, Istituti Clinici Scientifici Maugeri IRCCS, 27040 Montescano, Italy; monica.panigazzi@icsmaugeri.it (M.P.); elena.prestifilippo@icsmaugeri.it (E.P.); cesare.grilli@icsmaugeri.it (C.G.)

**Keywords:** polyneuropathy, rehabilitation, exergaming, biofeedback, cognitive function, quality of life, functional status, longitudinal

## Abstract

**Background:** Polyneuropathies impair sensory, motor, and autonomic functions, affecting functional status, cognition, and quality of life. This pilot study investigated the effects of exergame with biofeedback training (Riablo system) versus standard rehabilitation on these outcomes in outpatients with mixed-etiology polyneuropathies. **Methods:** Seventeen outpatients were assigned to standard rehabilitation (Group 1, n = 9) or combined standard plus Riablo training (Group 2, n = 8) over three weeks. Functional status, pain, cognition, quality of life, and psychological well-being were assessed pre- and post-intervention, with a 6-month follow-up. Outcome measures included the Morse Fall Scale, Visual Analogue Scales for pain and autonomy, Montreal Cognitive Assessment (MoCA), Trail Making Test (TMT), Stroop Test, Frontal Assessment Battery (FAB), Verbal fluency test, the Short-Form Health Survey-12 (SF-12), and the Patient Health Questionnaire-4 (PHQ-4). Longitudinal changes and between-group differences were analyzed using nonparametric statistics. **Results:** Both groups showed significant improvements in functional status and global cognition at post-intervention. Group 2 demonstrated greater improvements in executive functions and attention, with significant reductions in pain and fall risk. At 6-month follow-up, Group 2 maintained post-intervention gains in QoL and psychological outcomes, while Group 1 showed a significant decline. Technology evaluation revealed high usability and positive psychosocial impact in Group 2, with strong correlations between executive function improvements and device usability. **Conclusions:** Integrating exergames with biofeedback into standard rehabilitation may provide broader and longer-lasting benefits for polyneuropathy patients. These findings support further large-scale trials to confirm efficacy and optimize technology-assisted rehabilitation protocols.

## 1. Introduction

Polyneuropathies represent a heterogeneous group of peripheral nervous system disorders characterized by progressive deterioration of sensory, motor, and autonomic functions, significantly impacting patients’ functional status, cognitive performance, and quality of life [1,2]. The prevalence of polyneuropathies increases with age, affecting approximately 2–8% of the general population, with higher rates observed in individuals over 65 years [3,4]. They may arise from a wide range of etiologies, including metabolic conditions such as diabetes mellitus, autoimmune and inflammatory disorders, infections, toxic exposures (e.g., chemotherapy-induced neuropathy), nutritional deficiencies, and hereditary diseases [1,4]. These conditions often result in substantial functional limitations, including increased fall risk, chronic pain, reduced mobility, and decreased independence in the activities of daily living (ADLs) [5,6].

Traditional rehabilitation approaches for polyneuropathies have primarily focused on conventional physiotherapy and occupational therapy interventions, typically delivered in healthcare settings through scheduled sessions conducted one or more times per week (e.g., supervised strength, balance, and functional exercises aimed at improving mobility, safety, and independence in daily activities). However, emerging evidence suggests that technology-enhanced rehabilitation strategies may offer superior outcomes through increased patient engagement, real-time feedback, and personalized training protocols [7,8]. Exergaming, defined as exercise combined with video gaming technology, has shown promising results in various neurological conditions by improving motivation, adherence, and functional outcomes [9,10].

The integration of biofeedback mechanisms in rehabilitation protocols has gained considerable attention too, due to its potential to enhance motor learning and functional recovery. Biofeedback is a therapeutic technique in which sensors provide real-time feedback about bodily signals, allowing individuals to learn how to better control their movements and physiological responses. Biofeedback may also provide real-time information about physiological processes, enabling patients to develop greater awareness and control over their movements and bodily functions [11,12]. Recent evidence has highlighted the effectiveness of biofeedback-enhanced interventions in improving balance, gait parameters, and functional mobility in patients with neurological disorders [13,14].

Cognitive impairment is increasingly recognized as a significant comorbidity in patients with polyneuropathies, particularly affecting executive functions, attention, and processing speed [15]. These deficits, together with chronic pain, functional limitations, and a progressive disease course, substantially compromise quality of life (QoL) across physical, psychological, and social domains [16,17,18,19]. The relationship between peripheral neuropathy, cognitive dysfunction, and reduced QoL may be mediated by shared pathophysiological mechanisms, including inflammation, oxidative stress, and metabolic dysregulation [20]. Rehabilitation interventions that simultaneously target motor, cognitive, and psychological domains may therefore provide synergistic benefits, highlighting the importance of longitudinal assessment to understand sustained outcomes and identify factors contributing to long-term well-being. The Riablo system (CoRehab, Italy) represents an innovative rehabilitation platform that combines exergaming principles with biofeedback technology. This device provides interactive training scenarios that challenge balance, coordination, and cognitive functions while delivering real-time performance feedback [21]. Prior studies have suggested potential benefits of such integrated approaches [22,23,24,25,26], though comprehensive evaluation in polyneuropathy populations still lacks.

Despite the growing interest in technology-enhanced rehabilitation, there is a paucity of controlled studies examining the specific effects of exergames with biofeedback training in patients with polyneuropathies. Furthermore, most existing research has focused on short-term outcomes, typically assessed over intervention periods ranging from a few weeks to approximately three months, with limited investigation of longitudinal effects on functional, cognitive, and psychological domains. In contrast, the present study extends this perspective by examining outcomes over a longer follow-up period, allowing for a more comprehensive evaluation of sustained rehabilitation effects. Understanding the sustained impact of these interventions is crucial for clinical decision-making and resource allocation.

The present pilot study aimed to preliminarily investigate the effects of exergame with biofeedback training compared to standard rehabilitation on functional status, cognitive performance, and quality of life in outpatients with mixed-etiology polyneuropathies. Secondary objectives included assessment of rehabilitation experience, psychosocial impact of technology use, and perceived usability. It is expected that the integrated approach would demonstrate superior outcomes compared to standard rehabilitation alone, with sustained benefits observed at 6-month follow-up.

## 2. Materials and Methods

This study was approved by the Ethics Committee of the Istituti Clinici Scientifici (ICS) Maugeri IRCCS (March 2021, prot. n.2517CE) and was conducted in accordance with the Declaration of Helsinki. All participants provided written informed consent to participate in the study before enrollment. The full description of the study protocol was registered (ClinicalTrtials.gov ID: NCT05399043) and published elsewhere [27].

### 2.1. Study Design and Participants

A pilot prospective, two-arm, open-label, non-randomized study design was adopted. In a real-world rehabilitation setting (ICS Maugeri, Montescano Institute), consecutive enrollment was conducted. Patients were screened for eligibility at hospital admission by a multidisciplinary panel consisting of physical and rehabilitation medicine specialists, psychologists, nurses, occupational therapists, and researchers. Inclusion criteria were (i) 18 years of age or older; (ii) diagnosis of polyneuropathy requiring rehabilitation intervention (restrictions on the etiology were not applied); (iii) no severe clinical conditions (i.e., chronic heart failure NYHA-IV, ischemic heart disease CCS-IV, neoplastic diseases, or acute respiratory diseases); (iv) absence of severe cognitive impairment (Montreal Cognitive Assessment—MoCA score > 15.5 [28]); and (v) no language disorders (e.g., aphasia) or mental health conditions or psychiatric disorders, as assessed according to DSM-5-TR criteria, that could have compromised study participation or data reliability.

### 2.2. Intervention

Patients were assigned to two study groups based on their individualized rehabilitation program, which was determined according to routine multidisciplinary clinical practice where the study was conducted [29]. Importantly, while rehabilitation planning followed a patient-centered and individualized clinical approach, group allocation was based on predefined and standardized intervention schemes. All rehabilitation interventions were delivered and supervised by physical therapists with expertise in neurological and orthopedic treatment. Sessions were conducted in dedicated rehabilitation gyms within the hospital setting as part of routine outpatient care. Each patient’s recovery pathway was designed based on the integration of the International Classification of Diseases (ICD) and the International Classification of Functioning, Disability, and Health (ICF) models. Rehabilitation procedures were adapted following the patient’s specific diagnosis, disability severity, and recovery objectives, in favor of a personalized rehabilitation project and program, allowing for clinical tailoring within a fixed and comparable intervention framework across groups.

Patients assigned to Group 1 received standard treatment consisting of daily 90-min sessions of physical therapy over three weeks. All sessions were continuously supervised by the treating physical therapist, who provided instructions, ensured patient safety, and adjusted exercise difficulty as needed. Sessions included range of motion exercises (passive, active-assisted, and active), progressive resistive exercises, balance and strength training, proprioceptive training, and aerobic conditioning. Those assigned to Group 2 received both standard rehabilitation (60 min daily) and technology-enhanced training (30 min daily) with the support of the Riablo System over three weeks, resulting in an identical total daily rehabilitation duration (90 min) and overall dose as group 1. Thus, the two groups differed exclusively in the internal composition of the rehabilitation sessions, while treatment duration, frequency, and overall dose were maintained as comparable. For the technology-enhanced component, physical therapists assisted with device setup and supervised exergame-based biofeedback training. The Riablo^TM^ (CoRehab, Trento, Italy) is a medical device consisting of wearable sensors placed on patients’ limbs and a stabilometric platform that transmits motion data to software providing biofeedback and displaying digital games through a screen while exercising. The system monitors and trains limb range of motion, balance, and anterior-posterior and lateral flexion/extension of the spine with a sensor placeable in the lumbar area. The device was designed to address orthopedic and neuromotor conditions to facilitate passive and active mobility and proprioceptive capacity recovery. Patients’ adherence to each rehabilitation session was monitored on a daily basis by the clinical team responsible for patient care.

### 2.3. Outcome Measures

Following preliminary collection of socio-demographic (age, gender, marital status, living condition, and education) and clinical data (body mass index—BMI, comorbidities, and risk factors), all patients underwent pre-post intervention multi-domain evaluation.

Functional status was assessed with the Morse Fall Scale (MFS) [30], and a Visual Analogue Scale (VAS) was used to evaluate perceived pain (range: 0–10) and autonomy in ADLs (range: 0–100).

Cognitive functions were assessed through a comprehensive battery of neuropsychological tests, including the MoCA [28] for global cognition, the Trail Making Test (TMT-A and TMT-B) [31], the shortened version of the Stroop Colour Word Test [32], and the Frontal Assessment Battery (FAB) [33] for attention and executive functions, and the phonemic verbal fluency test [34] as a measure to evaluate language.

QoL and psychological status evaluation (pre- and post-intervention and 6-month follow-up) was conducted through validated self-report questionnaires. The Short-Form Health Survey-12 (SF-12) [35] was used to provide composite scores for physical (PCS) and mental (MCS) health, and the VAS scale of the Euro-QoL (EQ-VAS) [36] for overall health. Anxiety and depression symptoms were measured with the Patient Health Questionnaire-4 (PHQ-4) [37].

At post-intervention, all patients also provided an evaluation on their rehabilitation program through the Client-Centred Rehabilitation Questionnaire (CCRQ) [38], while only those assigned to Group 2 underwent technology evaluation with the Psychosocial Impact of Assistive Device Scale (PIADS) [39], the System Usability Scale (SUS) [40], and the Experience in Technology-based Rehabilitation Schedule (ExTR) (see Supplementary Materials for details). A detailed description of each outcome measure is provided in the original study protocol, which is published elsewhere [27].

### 2.4. Data Analysis

Descriptive statistics were calculated for all study variables with means and standard deviations for continuous variables and frequencies for categorical variables. Between-group differences at baseline were assessed using the chi-squared test or the Mann–Whitney U test to verify group comparability. Given the pilot nature of the study and the related small sample size, a non-parametric approach was applied to evaluate intervention effects. Within-group pre-post intervention changes on functional and cognitive outcomes were analyzed using Wilcoxon signed-rank tests, while Mann–Whitney U tests were performed to compare change scores (delta values) between the two study groups. Friedman tests were then conducted to estimate main effects of time on QoL and psychological outcomes across the three assessment timepoints (pre-, post-intervention, and 6-month follow-up). Post-hoc pairwise comparisons were performed using the Wilcoxon signed-rank test. Kendall’s coefficient of concordance (W) was estimated as an indicator of effect size for the Friedman tests. Finally, exploratory correlations were calculated between changes in rehabilitation outcomes (delta values), 6-month follow-up mean scores, and technology evaluation variables by Spearman’s (rho) rank coefficients. All statistical analyses were performed using SPSS v. 29.0. Statistical significance was set at *p* ≤ 0.05.

## 3. Results

### 3.1. Socio-Demographic and Clinical Characteristics of the Sample

A total of 17 participants were enrolled in this pilot study, with 9 patients allocated to Group 1 (standard rehabilitation) and 8 patients to Group 2 (standard rehabilitation + Riablo training). The baseline socio-demographic and clinical characteristics of the study sample are presented in Table 1.

The mean age of the total sample was 71.8 ± 9.8 years. The majority of participants were male (82.4%), married (64.7%), and living with others (70.6%). Educational background varied, with 47.1% having completed middle school education. The mean BMI was 26.5 ± 4.3 kg/m^2^, indicating slight overweight status in the sample.

Regarding clinical characteristics, 76.5% of participants had no significant comorbidities, while 23.5% presented with one comorbidity, primarily type-2 diabetes mellitus or a history of transient ischemic attack. Risk factors such as smoking behavior, dyslipidemia, arterial hypertension, hyperuricemia, disease familiarity, or past clinical events were present in 35.3% of the sample.

No statistically significant differences were observed between groups for any baseline socio-demographic and clinical characteristics, indicating adequate patient allocation and group comparability.

### 3.2. Pre-Post-Intervention Effects

Intervention effects on study outcomes are summarized in Table 2. Overall, both groups demonstrated significant widespread improvements following training completion. Notably, Group 2 showed significant changes in more cognitive domains compared to Group 1, with technology proving superior at specifically targeting patients’ executive functions. The results obtained for each outcome are described in detail.

#### 3.2.1. Functional Outcomes

Both groups showed significant improvements in functional status. The evaluation with the VAS (0–100) showed a mean improvement of 23.3 ± 13.2 points in Group 1 (*p* = 0.007) and a similar change of 24.3 ± 9.8 points in Group 2 (*p* = 0.017). Although no significant between-group differences were observed in the magnitude of change, Group 2 reported significantly higher scores at post-intervention evaluation (*p* = 0.042).

Perceived pain levels (VAS 0–10) showed significant reductions in both groups. Group 1 experienced a decrease of 1.9 ± 2.1 points (*p* = 0.041), while Group 2 demonstrated a reduction of 2.6 ± 1.9 points (*p* = 0.027). The between-group comparison of change scores was not significant (*p* = 0.470).

Fall risk showed minimal changes in both groups, with no statistically significant improvements observed. However, a significant intergroup comparison was found at training completion, suggesting Group 2 to be at lower risk of falls compared to Group 1 (*p* = 0.042).

#### 3.2.2. Cognitive Outcomes

Both groups reported significant changes in global cognition as measured by the MoCa (Group 1: 3.0 ± 3.4, *p* = 0.038; Group 2: 3.0 ± 2.7, *p* = 0.025), but no between-group differences were observed.

Attention and processing speed revealed differential effects between groups. While Group 1 showed minimum change, Group 2 showed a significant improvement (TMT-A: 17.8 ± 20.4, *p* = 0.036). However, no significant between-group comparisons were found.

Executive functions assessment revealed significant between-group differences in delta mean scores (TMT-B: *p* = 0.038). Although no significant within-group effects were estimated, Group 2 showed greater improvements (−30.2 ± 44.4, *p* = 0.093) compared to Group 1, which conversely exhibited a worsening in subtest scores (14.3 ± 37.3).

The results from the Stroop test indicated differential effects on inhibitory control. While error rates showed no significant changes in either group, processing time demonstrated significant improvements in Group 2 only (−6.9 ± 5.3, *p* = 0.017).

In conclusion, FAB scores significantly improved in both groups (Group 1: 1.8 ± 1.2, *p* = 0.012; Group 2: 2.2 ± 1.9, *p* = 0.028), although no significant differences emerged from group comparison.

Verbal fluency showed a numerical increase in both groups, with Group 2 reporting a larger improvement (6.6 ± 10.3) compared to Group 1 (0.7 ± 4.1), though neither reached statistical significance.

### 3.3. Rehabilitation Experience and Technology Evaluation

Rehabilitation experience evaluation with the CCRQ revealed no significant differences across all domains, indicating comparable satisfaction with both rehabilitation approaches. However, although non-significant, Group 2 generally reported higher mean scores than Group 1 (Table 3).

Informative evidence on technology evaluation was finally observed from Group 2. The PIADS mean scores indicated positive impacts across all three domains. Moreover, satisfactory ratings were found on technology experience of use (ExTR: 41.4 ± 12.8) and perceived usability (65.9 ± 21.2), ultimately suggesting good device acceptability.

### 3.4. Longitudinal Analysis (6-Month Follow-Up)

The longitudinal analysis revealed distinct patterns of change in QoL and psychological outcomes between the two groups (Table 4). These were analyzed independently through separate models of variance, as baseline mean scores differed significantly between the two groups, making the trends not comparable. Group 1 showed a significant main effect of time across almost all the outcomes investigated at 6 months, though indicating a progressive decrease over time. Conversely, Group 2 displayed more stable patterns following post-intervention improvements, presumably suggesting a greater long-lasting impact of technology.

**Figure 1:**
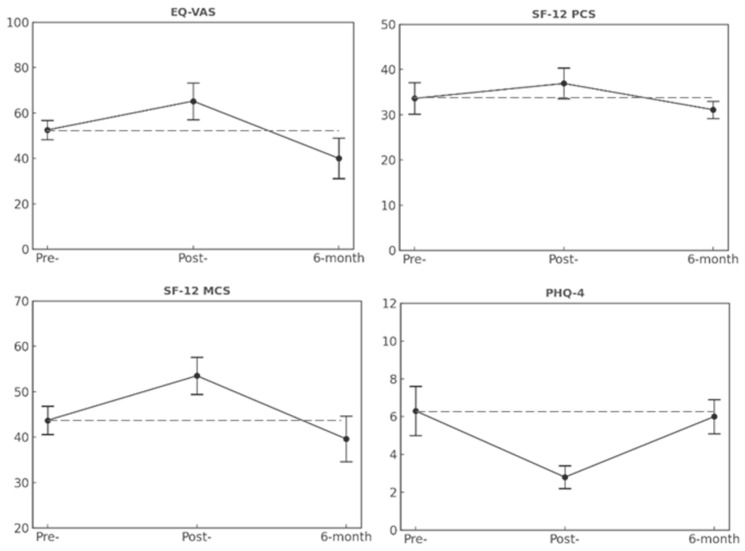
Longitudinal patterns on QoL and psychological outcomes in Group 1. Note. Error bars indicate standard errors. The dashed horizontal line represents the baseline (pre-) mean and is included as a reference to illustrate deviations at subsequent time points.

Figure 1 shows the longitudinal variations observed in Group 1 on EQ-VAS, SF-12 (physical and mental health components), and PHQ-4 mean scores. In detail, the results on EQ-VAS varied significantly over time (χ^2^ = 7.043, *p* = 0.030), with scores improving from baseline to post-intervention but returning to baseline levels at 6 months. The SF-12 MSC followed a similar pattern (χ^2^ = 6.333, *p* = 0.042), while the SF-12 PCS remained stable throughout the follow-up period. Likewise, PHQ-4 scores significantly changed over time (χ^2^ = 9.818, *p* = 0.007), with improvement observed at post-intervention that was not sustained at 6-month follow-up. The Wilcoxon signed-rank test was adopted as a non-parametric post-hoc procedure. Pairwise comparisons confirmed the trends observed through the analysis of variance, further supporting the presence of significant differences between post-intervention and 6-month follow-up timepoints.

**Figure 2:**
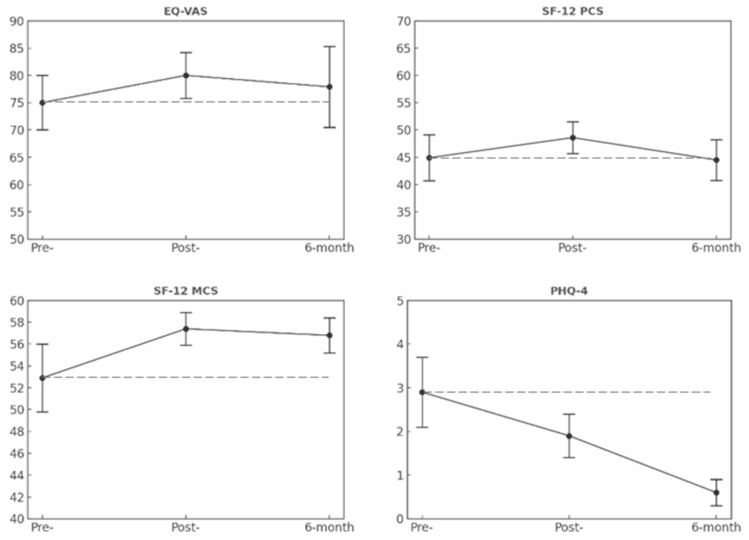
Longitudinal patterns of QoL and psychological outcomes in Group 2. Note. Error bars indicate standard errors. The dashed horizontal line represents the baseline (pre-) mean and is included as a reference to illustrate deviations at subsequent time points.

Figure 2 shows the longitudinal changes observed in Group 2. In contrast to Group 1, patients who participated in technology-enhanced training reported maintenance of post-intervention improvements across all study outcomes. Specifically, no significant main effects of time were found in any of the QoL outcomes. However, clinical improvements were observed from baseline to post-intervention evaluations, and stability through the 6-month follow-up was registered. Regarding the PHQ-4, Group 2 showed a progressive decrease in anxiety and depression symptoms over the study period (χ^2^ = 6.870, *p* = 0.032), with significant improvements observed at 6 months from both baseline and post-intervention assessments.

### 3.5. Correlations with Technology Evaluation

Table 5 shows the correlations explored in Group 2 between technology evaluation variables and rehabilitation outcomes. Specifically, significant positive relationships were found between executive function change (Δ FAB) and perceived device usability (SUS) and between verbal fluency improvements and the PIADS self-esteem subscale mean scores. A significant estimate was also found between the SF-12 mental health subcomponent measured at 6-month follow-up and the PIADS ability subscale. Notably, strong effects in all three correlations were observed.

## 4. Discussion

This pilot study contributes novel preliminary evidence on the sustained (six-month) benefits of biofeedback-enhanced exergaming in polyneuropathy rehabilitation—extending prior short-term findings by demonstrating superior long-term gains in executive cognition, fall risk reduction, and quality of life maintenance across integrated functional, cognitive, and psychological domains [9,10,11,12,13,14,22,23,24,25,26,41,42,43]. The findings indicate that both standard and technology-enhanced rehabilitation protocols can effectively provide improvements in comprehensive multi-domains covering functional, cognitive, and psychological outcomes. However, the addition of a biofeedback-driven system, such as Riablo, appears to confer specific advantages, particularly in executive and attentional cognitive domains, and in maintaining long-term improvements in QoL.

In our study, participants using the technological intervention had a mean age of 66.9 ± 11.6 years, which falls within the range where older adults generally benefit from technology-assisted care and rehabilitation. Literature indicates that outcomes are influenced more by functional status, cognitive profile, frailty, and psychosocial factors than by chronological age itself [44]. Rehabilitation studies further show that, although gains may be somewhat attenuated in the very old (≥85 years), meaningful improvements are still achieved, and age alone is not a sufficient reason to restrict access to intensive or technology-supported rehabilitation [45,46]. Overall, these findings suggest that similar technology-supported interventions are likely to be applicable and beneficial across a broad spectrum of older adults, provided they are adapted to age-related cognitive, physical, and usability needs [44,46].

From a functional perspective, participants demonstrated meaningful improvements following rehabilitation, regardless of the type of intervention. This aligns with prior evidence suggesting that rehabilitation can improve motor function and reduce disability in individuals with polyneuropathies and other neurological or neurodegenerative conditions [47,48]. A systematic review [41] has further supported the efficacy of exercise interventions, particularly in diabetic peripheral neuropathy, regarding balance, mobility, and nerve conduction measures. However, most available data focus on diabetic and chemotherapy-induced neuropathies, underscoring the need for high-quality studies in other neuropathy subtypes to refine clinical recommendations [41]. Nonetheless, patients in the technology-enhanced group of this pilot study achieved higher post-intervention functional scores and reported a lower risk of falls compared with those in the standard group. These findings mirror results from previous investigations, such as the study by Maranesi et al. [42] in Parkinson’s disease, showing that both conventional and technology-based interventions improve balance, yet only exergame-based rehabilitation yields significant reductions in fall risk. Similarly, a network meta-analysis of 52 randomized controlled trials demonstrated that exergaming with motion capture provides therapeutic benefits comparable to conventional exercise, with significant gains in functional mobility [43].

At the cognitive level, the present results suggest that exergame-based interventions are also effective in enhancing executive and attentional functions. This finding is consistent with the growing literature recognizing the cognitive benefits of technology-assisted rehabilitation [22,23,24,25,26]. The superior cognitive performance observed in the technology-enhanced group may be explained by the simultaneous engagement of motor execution, visuospatial processing, attention, and executive control required by exergame-based tasks. Mechanistically, exergaming and biofeedback-based rehabilitation may foster neuroplasticity through enriched environments that combine cognitive-motor complexity, real-time feedback, and motivational engagement to stimulate neural adaptation [7,8,16,49,50,51]. The continuous exposure to real-time feedback likely facilitates error detection, performance monitoring, and adaptive behavioral regulation, which are core components of executive functioning and actively engage prefrontal and frontal-parietal networks. Several studies have demonstrated that such approaches can reliably promote improvements in executive functioning and attention across diverse clinical populations [51,52,53,54]. Likewise, integrated rehabilitation models, combining conventional and technology-mediated training, have been associated with cognitive enhancement in various neurological disorders [22,23,24,25,26,55,56]. In this context, the present findings suggest that the added cognitive load and feedback-driven self-regulation inherent to exergame-based biofeedback training may explain the selective advantages observed in executive and attentional domains compared to standard rehabilitation alone. The current study extends these findings to individuals with polyneuropathies, who often experience comorbid motor and cognitive impairments [1,2]. Several outcomes also exhibited favorable numerical trends without reaching statistical significance, which may be attributable to limited statistical power related to the pilot design and small sample size.

Importantly, the durability of the functional and psychological improvements observed throughout the follow-up period aligns with existing literature indicating that technology-assisted rehabilitation produces not only immediate benefits but also sustained enhancements in mobility, psychological well-being, and quality of life over time. Prior investigations have reported high levels of patient acceptance of digital technologies, which contribute to functional improvements and reductions in anxiety symptoms [56]. Similarly, virtual-reality rehabilitation has demonstrated notable efficacy in ameliorating mental health outcomes, such as reducing depression and anxiety, in neurological populations compared to conventional therapy [57]. The prolonged impact of these interventions likely may arise from increased patient engagement, intervention adaptability, and motivation fostered by interactive and personalized training paradigms, as corroborated by our usability findings [7,8,16,48,58]. While most previous studies have predominantly focused on short-term effects, with limited longitudinal exploration across functional, cognitive, and psychological domains, these present findings contribute valuable preliminary evidence demonstrating sustained recovery trajectories over a six-month period. This underscores the critical importance of assessing extended outcomes to better guide clinical decision-making and healthcare resource allocation.

Regarding user experience, participants reported generally high satisfaction across both rehabilitation modalities. However, those exposed to the Riablo system rated their overall experience more positively, citing greater perceived benefits. The technology was well accepted and achieved high usability scores, with noted positive effects on adaptability and self-esteem as measured by standardized assistive technology scales [27]. These results reinforce the feasibility and acceptability of biofeedback-enhanced exergaming for the rehabilitation of this clinical population.

While this study provides promising preliminary evidence for the efficacy of integrating exergame biofeedback technology in polyneuropathy rehabilitation over both the short term and a six-month period, several important limitations must be acknowledged. First, the non-randomized design and small sample size limit the generalizability and statistical power of the findings, as is common in early-phase pilot studies. Larger, randomized controlled trials that include diverse patient populations are necessary to confirm these results and identify subgroups most likely to benefit. Determining the appropriate “dose” of exergaming and biofeedback training—balancing intensity and patient tolerance—is critical to maximizing neuroplasticity and functional gains without causing fatigue or disengagement. Likewise, understanding the timing and sequencing of integrating such technologies into standard rehabilitation pathways is essential to facilitate adoption and ensure sustained benefits [54]. Also, no structured or standardized rehabilitation program was prescribed after discharge, leading to no formal monitoring of rehabilitation behaviors, which may limit control over potential confounding influences on long-term outcomes. Moreover, robust independent evidence is essential to address the possible threat of the so-called “decline effect”—the observed pattern in which early promising results from pioneer studies often diminish as larger, independent trials yield more conservative outcomes [59].

Second, although the intervention demonstrated favorable usability and psychosocial impacts, broader challenges remain for clinical translation and implementation. Factors such as digital literacy, technical support availability, integration within multidisciplinary teams, and cost-effectiveness must be rigorously addressed. Without establishing standardized protocols and clear guidelines for technology deployment, rehabilitation teams risk increased expenses and resource burdens that may undermine the overall value of the intervention, both in this and other clinical populations [48,54,58].

Therefore, future research should prioritize delineating evidence-based protocols that specify the optimal dosage, timing, and procedural integration of technological tools like Riablo within personalized rehabilitation plans. Cost-benefit analyses and implementation science approaches will be key to informing sustainable adoption in clinical practice while minimizing unnecessary expenditures. Addressing these gaps systematically will help translate the promising potential of technology-enhanced rehabilitation into scalable and effective solutions for patients with polyneuropathies and other complex neurological disorders.

## 5. Conclusions

Given the substantial burden polyneuropathies impose on physical, cognitive, and emotional dimensions, the preliminary findings of this pilot study advocate for a paradigm shift favoring multimodal, technology-assisted rehabilitation approaches for polyneuropathy patients. Although large-scale randomized controlled trials are needed, these findings suggest that the integration of exergaming and biofeedback with the Riablo system into a standard rehabilitation program for this clinical population resulted in broader and more sustained improvements, particularly in executive cognitive domains and psychological well-being, compared to traditional rehabilitation alone. Moreover, this approach seems to be well tolerated, perceived as useful by participants, and associated with lasting effects on QoL and emotional status up to six months after treatment.

## Figures and Tables

**Table 1:** Baseline socio-demographic and clinical characteristics of the study sample (n = 17).

Variables	Group 1	Group 2	Total	*p*
Age, Mean ± SD	76.2 ± 5.3	66.9 ± 11.6	71.8 ± 9.8	0.139
Gender, n (%)				
*Male*	7 (77.8)	7 (87.5)	14 (82.4)	0.600
*Female*	2 (22.2)	1 (12.5)	3 (17.6)	
Marital Status, n (%)				0.402
*Single/Separated/widowed*	4 (44.4)	2 (25.0)	6 (35.3)	
*Married*	5 (55.6)	6 (75.0)	11 (64.7)	
Living condition, n (%)				0.707
*Alone*	3 (33.3)	2 (25.0)	5 (29.4)	
*With others*	6 (66.7)	6 (75.0)	12 (70.6)	
Education, n (%)				0.325
*None or primary*	4 (44.5)	1 (12.5)	5 (29.4)	
*Middle school*	3 (33.3)	5 (62.5)	8 (47.1)	
*High school or higher*	2 (22.2)	2 (25.0)	4 (23.5)	
BMI, Mean ± SD	25.5 ± 4.9	28.1 ± 3.0	26.5 ± 4.3	0.142
Comorbidity, n (%) *				0.200
*None*	8 (88.9)	5 (62.5)	13 (76.5)	
*One*	1 (11.1)	3 (37.5)	4 (23.5)	
Risk Factors, n (%) °				0.232
*None*	7 (77.8)	4 (50.0)	11 (64.7)	
*One or more*	2 (22.2)	4 (50.0)	6 (35.3)	

Notes. *p*-values are from the chi-squared test and Mann–Whitney test for categorical and continuous variables, respectively. BMI, Body Mass Index; SD, Standard deviation; * Comorbidities include type-2 diabetes mellitus and transient ischemic attack; ° Risk factors include smoking behavior, dyslipidemia, arterial hypertension, hyperuricemia, disease familiarity, and past clinical events.

**Table 2:** Intervention effects on functional and cognitive outcomes.

Variables	Group 1 (n = 9)				Group 2 (n = 8)				
	Pre-	Post-	Δ	*p*	Pre-	Post-	Δ	*p*	*p*Δ
Functional									
*MFS*	19.4 ± 10.1	21.1 ± 7.4 *	−1.7 ± 5.0	0.317	11.3 ± 6.9	10.7 ± 7.3 *	0.6 ± 0.4	1.00	0.758
*VAS Functional status (0–100)*	40.6 ± 15.5	63.9 ± 17.3 *	23.3 ± 13.2	0.007	57.9 ± 16.8	82.1 ± 14.1 *	24.3 ± 9.8	0.017	0.681
*VAS Pain (0–10)*	3.8 ± 2.9	1.9 ± 1.6	−1.9 ± 2.1	0.041	4.4 ± 2.8	1.9 ± 1.8	−2.6 ± 1.9	0.027	0.470
Cognitive ^1^									
*MoCA*	24.9 ± 2.9	27.9 ± 1.3	3.0 ± 3.4	0.038	25.5 ± 3.5	28.5 ± 1.4	3.0 ± 2.7	0.025	0.743
*TMT-A*	30.2 ± 18.8	27.5 ± 21.1	0.5 ± 16.9	0.889	34.1 ± 19.7	16.3 ± 8.9	−17.8 ± 20.4	0.036	0.105
*TMT-B*	98.8 ± 62.7	113.1 ± 82.0	14.3 ± 37.3	0.176	89.9 ± 48.3	59.7 ± 26.6	−30.2 ± 44.4	0.093	0.038
*Stroop Errors*	2.7 ± 6.9	−0.2 ± 1.3	−2.9 ± 6.9	0.173	0.5 ± 1.5	−0.1 ± 0.3	−0.7 ± 1.5	0.225	0.481
*Stroop Time*	17.0 ± 14.9	15.8 ± 13.7	−1.2 ± 12.9	0.767	16.8 ± 4.8	9.8 ± 5.9	−6.9 ± 5.3	0.017	0.074
*FAB*	15.2 ± 2.4	17.0 ± 1.7	1.8 ± 1.2	0.012	15.1 ± 1.9	17.3 ± 0.9	2.2 ± 1.9	0.028	0.481
*Verbal fluency*	37.9 ± 8.9	38.6 ± 8.3	0.7 ± 4.1	0.722	38.0 ± 9.6	44.7 ± 11.8	6.6 ± 10.3	0.093	0.167

Notes. Values are means ± standard deviations; ^1^ Raw scores are adjusted for age and education; Δ, the difference (delta) between pre- and post-intervention values; and *p*-values are from within-group comparisons (Wilcoxon signed-rank test). * Significant between-group differences at post-intervention (Mann-Whitney test). *p*Δ values are from the Mann–Whitney test comparing delta mean scores between the two study groups. MFS, Morse Fall Scale; VAS, Visual Analogue Scale; MoCA, Montreal Cognitive Assessment; TMT, Trail Making Test; FAB, Frontal Assessment Battery.

**Table 3:** Perceived rehabilitation experience, technology psychosocial impact, experience of use, and usability.

Variables	Group 1	Group 2	*p*
CCRQ			
*Client Participation (range: 6–30)*	26.1 ± 7.9	28.8 ± 1.8	0.743
*Client-Centered Education (range: 5–25)*	19.8 ± 6.6	19.9 ± 2.9	0.481
*Outcome Evaluation (range: 4–20)*	17.1 ± 5.3	19.0 ± 1.6	0.541
*Family Involvement (range: 5–25)*	10.1 ± 11.6	7.5 ± 11.3	0.673
*Emotional Support (range: 5–25)*	17.4 ± 5.4	19.4 ± 1.4	0.673
*Physical Comfort (range: 4–20)*	17.0 ± 4.8	19.9 ± 0.4	0.093
*Continuity/Coordination (range: 5–25)*	17.1 ± 5.6	18.8 ± 3.7	0.606
PIADS			
*Ability (range: −3/+3)*	-	1.8 ± 0.7	
*Adaptability (range: −3/+3)*	-	2.2 ± 0.9	
*Self-esteem (range: −3/+3)*	-	1.5 ± 0.8	
ExTR (range: 0–52)	-	41.4 ± 12.8	
SUS (range: 0–100)	-	65.9 ± 21.2	

Notes: *p*-values are from the Mann–Whitney test. CCRQ, Client-Centred Rehabilitation Questionnaire; PIADS, Psychosocial Impact of Assistive Devices Scale; ExTR, Experience in Technological Rehabilitation Schedule; SUS, System Usability Scale.

**Table 4:** Quality of life and psychological status over 6 months and within-group effects.

				Friedman Test		
Group 1	Pre-	Post-	6-month	χ2	*p*	W
*EQ-VAS*	52.5 ± 4.2 *	65.2 ± 8.1 *°	40.0 ± 8.9 °	7.043	0.030	0.587
*SF-12 PCS*	33.6 ± 3.5	36.9 ± 3.4	31.1 ± 1.9	1.333	0.513	0.111
*SF-12 MCS*	43.7 ± 3.1 *	53.5 ± 4.1 *°	39.6 ± 5.0 °	6.333	0.042	0.528
*PHQ-4*	6.3 ± 1.3 *	2.8 ± 0.6 *°	6.0 ± 0.9 °	9.818	0.007	0.818
Group 2						
*EQ-VAS*	75.0 ± 5.0	80.0 ± 4.2	77.9 ± 7.4	3.176	0.204	0.227
*SF-12 PCS*	44.9 ± 4.2	48.6 ± 2.9	44.5 ± 3.7	2.889	0.236	0.206
*SF-12 MCS*	52.9 ± 3.1	57.4 ± 1.5	56.8 ± 1.6	1.407	0.495	0.101
*PHQ-4*	2.9 ± 0.8 °	1.9 ± 0.5 *	0.6 ± 0.3 *°	6.870	0.032	0.491

Values are means ± standard errors. *° Post-hoc within-group comparisons are significant from the Wilcoxon signed-rank test. W, Kendall’s coefficient.

**Table 5:** Correlations between study outcomes, pre-post intervention variations, 6-month follow-up evaluation, and technology psychosocial impact, experience of use, and usability.

Variables	PIADS			ExTR	SUS
	Ability	Adaptability	Self-Esteem		
MFS (Δ)	-	-	-	-	-
VAS Functional status (Δ)	−0.191	−0.179	−0.047	−0.467	−0.170
VAS Pain (Δ)	−0.046	−0.209	−0.109	0.468	0.564
MoCA (Δ)	0.482	−0.301	−0.012	−0.337	0.000
TMT-A (Δ)	0.169	0.470	0.551	0.386	0.323
TMT-B (Δ)	−0.108	0.012	0.395	0.048	0.443
Stroop Error (Δ)	0.037	−0.025	−0.196	−0.136	0.552
Stroop Time (Δ)	0.060	0.325	0.275	0.108	0.347
FAB (Δ)	−0.193	−0.133	−0.048	0.349	0.862 **
Verbal Fluency (Δ)	0.036	−0.181	0.790 *	0.084	0.287
6-month follow-up					
EQ-VAS	0.340	0.561	0.206	0.340	−0.168
SF-12 PCS	0.727	0.631	0.198	−0.073	−0.432
SF-12 MCS	0.782 *	0.072	0.090	0.436	0.000
PHQ-4	−0.152	−0.603	0.060	0.030	0.633

Δ, Delta values are calculated from the difference between post- and pre-intervention mean scores. Values are Spearman’s Rho correlation coefficients. *, *p* < 0.05; **, *p* < 0.01.

## Data Availability

Data are available from corresponding author upon request due to privacy and ethical restrictions.

## Supplementary Materials

The following supporting information can be downloaded at: https://www.mdpi.com/article/10.3390/brainsci16010045/s1, S1: Experience in Technological Rehabilitation Schedule (ExTR).
