# Supplementary material for: Effects of Exergame with Biofeedback Training on Functional Status, Cognition, and Quality of Life in Outpatients with Polyneuropathies: A Longitudinal Pilot Study
Source: Brain Sci. 2025 Dec 29;16(1):45. doi: 10.3390/brainsci16010045 (PMC12839407; doi:10.3390/brainsci16010045)
Supplement: Supplementary file 1 [file brainsci-4063966-supplementary.pdf › brainsci-4063966-supplementary.pdf]

Istituti Clinici Scientifici Maugeri

Experience in Technological Rehabilitation Schedule (ExTR)

Technological device name ______________________________

	Not at all	Slightly	Moderately	Very much	Extremely
1. Did you find the device easy to use?	o	o	o	o	o
2. Was it easy to accept the device as part of your rehabilitation program?	o	o	o	o	o
3. Did you find the device useful?	o	o	o	o	o
4. Did the device address your rehabilitation needs?	o	o	o	o	o
5. Would you use the device in the future?	o	o	o	o	o
6. Would you recommend using the device to others with a similar health condition?	o	o	o	o	o
7. While using the device, did you experience:					
a. General discomfort	o	o	o	o	o
b. Nausea	o	o	o	o	o
c. Dizziness	o	o	o	o	o

8. How did you find the device?

a. Boring	o	o	o	o	o	Fun
b. Difficult	o	o	o	o	o	Easy
c. Ineffective	o	o	o	o	o	Effective
d. Dangerous	o	o	o	o	o	Safe

Additional comments:

____

____

____

____

____

____

____
